# Changes of pulse wave transit time after haemodynamic manoeuvres in healthy adults: a prospective randomised observational trial (PWTT volunteer study)

**DOI:** 10.1016/j.bjao.2024.100291

**Published:** 2024-06-24

**Authors:** Johannes M. Wirkus, Fabienne Goss, Matthias David, Erik K. Hartmann, Kimiko Fukui, Irene Schmidtmann, Eva Wittenmeier, Gunther J. Pestel, Eva-Verena Griemert

**Affiliations:** 1 Department of Anaesthesiology, University Medical Center of the Johannes Gutenberg-University, Mainz, Germany; 2 Department of Anaesthesiology, Marienhaus Hospital, Mainz, Germany; 3 Institute of Medical Biostatistics, Epidemiology and Informatics (IMBEI), University Medical Center of the Johannes Gutenberg-University, Mainz, Germany

**Keywords:** fluid responsiveness, fluid resuscitation, haemodynamic monitoring, pulse wave transit time, stroke volume assessment

## Abstract

**Background:**

Pulse wave transit time (PWTT) shows promise for monitoring intravascular fluid status intraoperatively. Presently, it is unknown how PWTT mirrors haemodynamic variables representing preload, inotropy, or afterload.

**Methods:**

PWTT was measured continuously in 24 adult volunteers. Stroke volume was assessed by transthoracic echocardiography. Volunteers underwent four randomly assigned manoeuvres: ‘Stand-up’ (decrease in preload), passive leg raise (increase in preload), a ‘step-test’ (adrenergic stimulation), and a ‘Valsalva manoeuvre’ (increase in intrathoracic pressure). Haemodynamic measurements were performed before and 1 and 5 min after completion of each manoeuvre. Correlations between PWTT and stroke volume were analysed using the Pearson correlation coefficient.

**Results:**

‘Stand-up’ caused an immediate increase in PWTT (mean change +55.9 ms, *P*-value <0.0001, 95% confidence interval 46.0–65.7) along with an increase in mean arterial pressure and heart rate and a drop in stroke volume (*P*-values <0.0001). Passive leg raise caused an immediate drop in PWTT (mean change −15.4 ms, *P*-value=0.0024, 95% confidence interval −25.2 to −5.5) along with a decrease in mean arterial pressure (*P*-value=0.0052) and an increase in stroke volume (*P*-value=0.001). After 1 min, a ‘step-test’ caused no significant change in PWTT measurements (*P*-value=0.5716) but an increase in mean arterial pressure and heart rate (*P*-values <0.0001), without changes in stroke volume (*P*-value=0.1770). After 5 min, however, PWTT had increased significantly (*P*-value <0.0001). Measurements after the Valsalva manoeuvre caused heterogeneous results.

**Conclusion:**

Noninvasive assessment of PWTT shows promise to register immediate preload changes in healthy adults. The clinical usefulness of PWTT may be hampered by late changes because of reasons different from fluid shifts.

**Clinical trial registration:**

German clinical trial register (DRKS, ID: DRKS00031978, https://www.drks.de/DRKS00031978).

Flow-based haemodynamic monitoring techniques have generated interest in perioperative fluid management because they are minimally invasive. Using the oesophageal Doppler monitoring device, a flexible ultrasound probe is inserted orally or nasally to measure blood flow velocity in the descending aorta.^1^ Several clinical studies in various patient populations and two comprehensive meta-analyses^2^^,^^3^ have demonstrated improved patient-relevant outcomes, such as reduced hospital length of stay or reduced perioperative complication rates, when using oesophageal Doppler monitoring for intraoperative fluid management. Suprasternal Doppler ultrasound monitoring has shown less convincing results.^4^

A different approach to flow-based haemodynamic monitoring is the measurement of pulse wave transit time (PWTT). Measuring PWTT requires only basic monitoring, such as ECG and pulse oximetry. PWTT is the time needed for blood to be transported from the heart to the periphery and consists of three components^5^: the pre-ejection period, the transit time of the pulse wave through the elastic arteries, and finally the transit time through peripheral arteries. The pre-ejection period is the time from ventricular depolarisation to the onset of left ventricular ejection. The pre-ejection period decreases with increasing preload and is minimally affected by afterload or contractility.^6^^,^^7^ In a tilt-table study^8^ in healthy subjects, the pre-ejection period reflected fluctuations in central blood volume, while blood pressure remained unchanged, and PWTT reflected variations in the pre-ejection period. These findings could be reproduced during exercise stress tests.^9^ Studying patients requiring renal replacement therapy, PWTT was shown to correlate better with systolic blood pressure than pre-ejection period.^10^ Experimental investigations in both canine^11^ and porcine^12^ models have demonstrated that respiratory variations in PWTT (ΔPWTT) predict fluid responsiveness. These results were confirmed in a clinical study involving 38 elective abdominal surgical patients.^13^

Thus, PWTT is a noninvasive monitoring variable that shows promise for the prediction of fluid responsiveness intraoperatively. It has been known for decades that the velocity of the pulse wave is capable of efficiently reflecting cardiovascular function.^14^ It is also known that a ‘variety of factors in health and disease’^14^ may impact the velocity of the pulse wave. Currently unknown is the capability of the PWTT to mirror haemodynamic changes caused by changes in preload, inotropy, or afterload - or a combination of these.

This prospective observational study in adult healthy volunteers tests the effects on PWTT of different manoeuvres (‘stand-up test’ to induce venous pooling, ‘passive leg raise test’ to induce autotransfusion, ‘step test’ to mimic sympathetic activation, and ‘Valsalva manoeuvre’ to simulate an increase in intrathoracic pressure) representing specific components of haemodynamic variation.

## Methods

### Subjects

With approval of the State Ethics Committee of Rhineland-Palatinate, Germany (26 July 2018, authorisation number 837.254.14 [9493-F]) and written informed consent, 24 healthy adult volunteers were enrolled at the Department of Anaesthesiology of the University Medical Center Mainz, Germany. Exclusion criteria were cardiovascular diseases (in particular cardiac valve disease, hypotension, or taking of any cardiovascular drugs), acute illness within 7 days before measurements and expected difficulties in transthoracic echocardiography measurements (e.g. morbid obesity) or peripheral vasoconstriction secondary to hypothermia, drugs, or Raynaud syndrome.

The study plan was in accordance with the Declaration of Helsinki 1996 and International Council for Harmonisation (ICH) guideline E6 (Good Clinical Practice). The study was registered at the German Clinical Trials Register (DRKS, ID: DRKS00031978, https://www.drks.de/DRKS00031978). A per-protocol analysis including only subjects who had completed the entire study was performed.

This manuscript adheres to the Strengthening the Reporting of Observational Studies in Epidemiology (STROBE) guidelines.^15^

### Measurements

All study participants were monitored noninvasively with ECG, pulse oximetry, and noninvasive blood pressure measurements (LifeScope® Model J BSM-9101 Nihon Kohden Europe GmbH, Rosbach, Germany). In addition, baseline measurements of PWTT, cardiac output, and perfusion index were taken as follows.

### Pulse wave transit time

PWTT was assessed noninvasively with a six-lead ECG and a pulse oximeter (LifeScope® Model J BSM-9101). The beginning of the PWTT was defined by the R-wave of the ECG, and the end of PWTT was assessed by the upstroke of the plethysmography wave of a pulse oximeter attached to the earlobe. All PWTT measurements were corrected to a heart rate of 60 beats min^−1^.^16^ Pulse oximetry and ECG waveforms were recorded with sampling periods of 8 ms and 4 ms, respectively. The rising point of the plethysmography waveform representing pulse wave arrival was defined as the point where the differentiated signal reached 30% of the peak value of the derivative.^17^

### Cardiac output

Cardiac output was calculated using transthoracic echocardiography (Philips Sparq Ultrasound, Philips, Bothell, WA, USA). First, the diameter of the left ventricular outflow tract (LVOT_d_) was measured, displaying the parasternal long axis of the heart during systole. Second, displaying a five-chamber view of the heart, the velocity time integral of LVOT (LVOT_VTI_) was measured using a pulse wave Doppler. LVOT_d_ and LVOT_VTI_ values were assessed by calculating the mean of three consecutive measurements. Finally, cardiac output was calculated by:

CO=stroke volume (SV)×heart rate (HR), where SV=π×(LVOT_d_/2)^2^×LVOT_VTI_

### Perfusion index

Perfusion Index was assessed continuously by an additional pulse oximeter attached to the little finger of the non-dominant hand (Radical-7® Masimo Corp., Irvine, CA, USA). Perfusion index is calculated by comparing the pulsatile signal of the pulse oximeter to the non-pulsatile signal: perfusion index [%]=(AC/DC)×100, where AC (arterial compartment) is the pulsatile absorbance of the emitted light and DC (direct current) is the non-pulsatile absorbance.^18^

### Manoeuvres

After baseline measurements, volunteers underwent four randomly assigned manoeuvres: (1) stand-up test, (2) passive leg raise test, (3) step test, and (4) Valsalva manoeuvre.

To simulate venous pooling, the ‘*stand-up test*’ was carried out by standing up quickly from the supine position. Thus, blood is redistributed to the splanchnic bed and to the lower extremities, followed by an increase in heart rate and stroke volume to restore cardiac output and blood pressure.^19^

To simulate an increase in intravascular volume, the ‘*passive leg raise test*’ manoeuvre was carried out. From a semi-recumbent position with the trunk at 45° to mobilise additional blood volume, a passive elevation of the legs to a level of 45° with the trunk in the supine position was performed.^20^ Finally, volunteers were brought back to the semi-recumbent position. Great care was taken to communicate the required postural changes in advance.

To simulate adrenergic activation, a 3-min ‘*step-test*’ was carried out by climbing a chair of 30 cm height, always starting with the same leg.^21^ Step frequency was determined by a metronome set to 96 steps per minute.

To simulate an increase in intrathoracic pressure, a ‘*Valsalva manoeuvre*’^22^ was carried out by instructing volunteers to blow into a tube for 30 s and to maintain a pressure of 30 mm Hg controlled by a manometer.

After completion of the manoeuvres, measurements of all monitored haemodynamic variables, including PWTT and cardiac output, were carried out after 1 and 5 min. The 5-min measurement after the ‘passive leg raise test’ was omitted, as the maximal effect of haemodynamic changes occurs within 1 min.^20^ Measurements during the ‘Valsalva manoeuvre’ were carried out after 15 and 30 s, respectively. In all volunteers, a final measurement was carried out in the supine position.

### Statistics

This study is an open, prospective, single-centre, randomised observational trial. To compare the different manoeuvres, a Williams crossover design was used with each volunteer serving as her or his own control.^23^ A Williams crossover design is supposed to be well balanced for carry-over effects.^24^ Assuming a normal distribution of measured values for haemodynamic measurements before and after each manoeuvre, a number of 18 volunteers would have been sufficient to obtain 95% confidence intervals for differences that have a half-width of at most 0.5 standard deviation (sd) with probability of at least 95%. However, to obtain a complete Williams crossover design, each possible sequence (4×3×2×1) of manoeuvres needs to be applied once; thus, 24 volunteers had to be included. Each of them was assigned at random to one sequence of manoeuvres as specified by the sealed envelope technique prepared by the Clinical Research Center of the Department of Anaesthesiology in advance.

#### Primary endpoint

Changes in PWTT measurements compared with baseline were assessed by descriptive analyses of the mean and sd for all measurements.

#### Secondary endpoint

Changes in stroke volume, heart rate, and mean arterial pressure after 1 and 5 min after manoeuvres and at the end of the study were assessed by descriptive analyses of mean and sd for all measurements. Mean changes in all haemodynamic variables investigated were compared using a linear mixed model to test for statistically significant changes induced by the manoeuvres. Additionally, a type III test of fixed effects to check for period effects and interaction of period and manoeuvre was carried out.

SAS 9.4 (SAS Institute Inc., Cary, NC, USA) was used for all statistical analyses.

## Results

Unless otherwise indicated, the values are presented as the mean (sd): the age of the volunteers was 22 – 55 yr, the height was 175.0 (10.6) cm, the weight was 70.1 (13.1) kg, and the BMI was 26.7 (2.2) kg m^−2^. Twelve of the 24 participants were female. Data from all 24 volunteers were analysed.

### Change in PWTT measurements after manoeuvres

**Fig 1:**
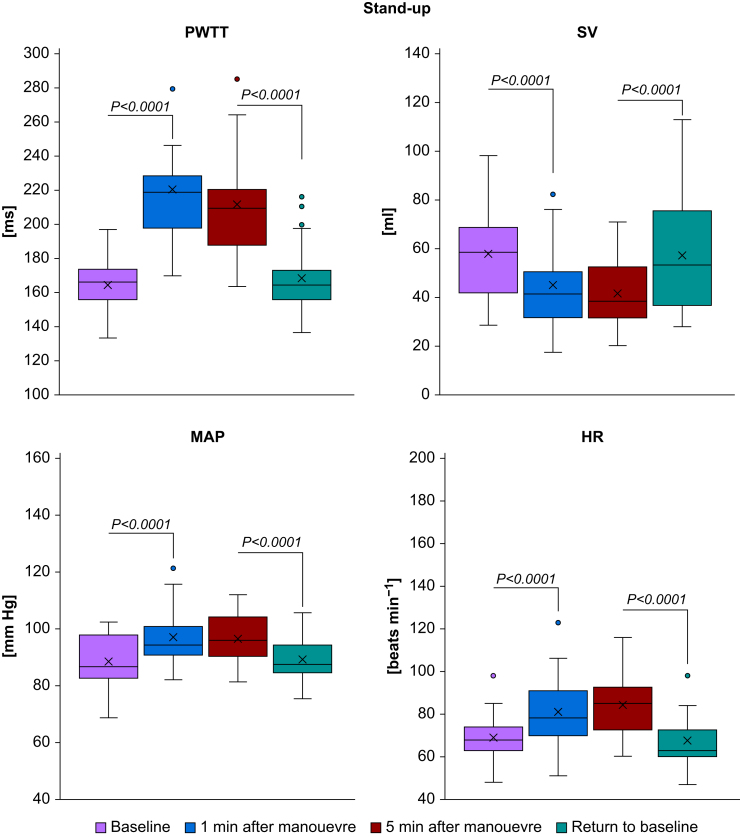
Boxplots of haemodynamic measurements during stand-up. HR, heart rate (beats min^−1^); MAP, mean arterial pressure (mm Hg); PWTT, pulse wave transit time (ms); SV, stroke volume (ml). Significant changes during manoeuvres are indicated by brackets and respective *P*-values.

**Fig 2:**
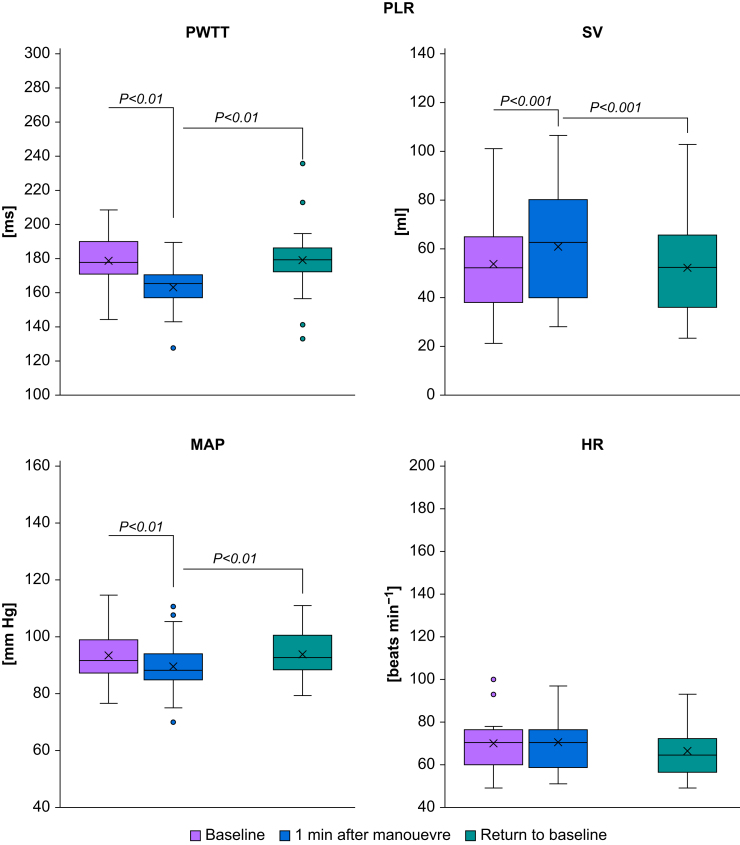
Boxplots of haemodynamic measurements during passive leg raise (PLR). HR, heart rate (beats min^−1^); MAP, mean arterial pressure (mm Hg); PWTT, pulse wave transit time (ms); SV, stroke volume (ml). Significant changes during manoeuvres are indicated by brackets and respective *P*-values.

**Fig 3:**
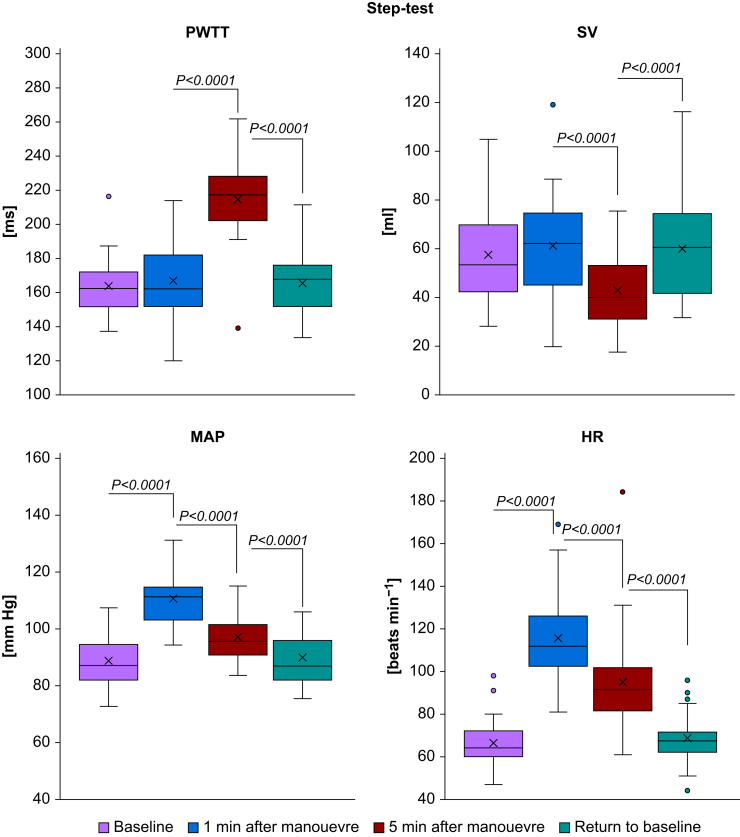
Boxplots of haemodynamic measurements during the step-test. HR, heart rate (beats min^−1^); MAP, mean arterial pressure (mm Hg); PWTT, pulse wave transit time (ms); SV, stroke volume (ml). Significant changes during manoeuvres are indicated by brackets and respective *P*-values.

**Fig 4:**
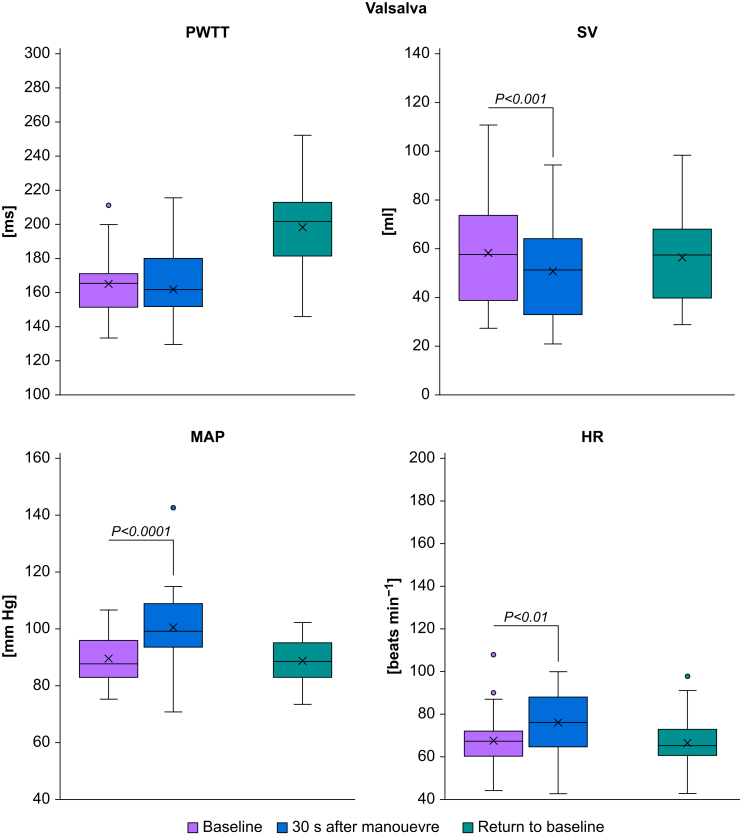
Boxplots of haemodynamic measurements during the Valsalva manoeuvre. HR, heart rate (beats min^−1^); MAP, mean arterial pressure (mm Hg); PWTT, pulse wave transit time (ms); SV, stroke volume (ml). Significant changes during manoeuvres are indicated by brackets and respective *P*-values.

‘Stand-up’ caused an immediate increase in PWTT (from 164.5 [16.6] to 220.4 [37.4] ms, *P*-value <0.0001), ‘passive leg raise’ caused an immediate decrease in PWTT (from 178.7 [15.6] to 163.4 [13.3] ms, *P*-value=0.0024), and the ‘step-test’ caused a deferred increase in PWTT (from 166.7 [22.7] to 214.8 [24.0] ms, *P*-value <0.0001). For details, see Table 1 and Fig 1, Fig 2, Fig 3, Fig 4. Measurements after the Valsalva manoeuvre were too heterogeneous to be interpreted (data not shown). A type III test for fixed effects ruled out period effects on PWTT and the interaction of manoeuvre and period.

**Table 1:** Pulse wave transit time (PWTT) measurements (ms) before and after manoeuvres. Mean and standard deviation (sd) of PWTT measurements (ms) before and after manoeuvres and respective *P*-values for changes between manoeuvres. T_0_=baseline before the start of the manoeuvre, T_1_=1 min after the completion of the manoeuvre (Valsalva: T_1_=30 s after start of manoeuvre), T_2_=5 min after the completion of the manoeuvre, T_end_=return to baseline. PLR, passive leg raise.

Manoeuvre	T_o_	T_1_	T_2_	T_end_
	Mean (sd)	Mean (sd)	Mean (sd)	Mean (sd)
**Stand-up**	164.5 (16.6)	220.4 (37.4)	212.0 (28.1)	168.6 (20.7)
		*P*<0.0001 (T_1_*vs* T_0_)	*P*=0.0961 (T_2_*vs* T_1_)	*P*<0.0001(T_end_*vs* T_2_) *P*=0.4210 (T_end_*vs* T_0_)
**PLR**	178.7 (15.6)	163.4 (13.3)	—	179.1 (21.0)
		*P*=0.0024 (T_1_*vs* T_0_)	—	*P*=0.0019 (T_end_*vs* T_1_) *P*=0.9462 (T_end_*vs* T_0_)
**Step-test**	163.9 (18.2)	166.7 (22.7)	214.8 (24.0)	165.6 (18.1)
		*P*=0.5716 (T_1_*vs* T_0_)	*P*<0.0001 (T_2_*vs* T_1_)	*P*<0.0001 (T_end_*vs* T_2_) *P*=0.7253 (T_end_*vs* T_0_)
**Valsalva**	165.1 (18.4)	162.0 (31.9)	—	166.7 (20.6)
		*P*=0.5442 (T_1_*vs* T_0_)	—	*P*=0.3472 (T_end_*vs* T_1_) *P*=0.7384 (T_end_*vs* T_0_)

### Change in haemodynamic measurements after the manoeuvres

‘Stand-up’ caused an immediate increase both in MAP (from 88.5 [9.0] to 97.0 [9.6] mm Hg, *P*-value <0.0001) and in heart rate (from 69.0 [12.0] to 81.3 [16.3] beats min^−1^, *P*-value <0.0001) and a decrease in stroke volume (from 58.1 [19.2] to 43.9 [17.1] ml, *P*-value <0.0001). ‘Passive leg raise’ caused an immediate decrease in MAP (from 93.6 [9.0] to 89.6 [9.9] mm Hg, *P*-value=0.0052) and an increase in stroke volume (from 53.5 [19.5] to 60.8 [23.2] ml, *P*-value=0.001), while heart rate remained unchanged (*P*=0.8903). The ‘step-test’ caused an increase both in MAP (from 88.9 [9.5] to 110.5 [9.2] mm Hg, *P*-value <0.0001) and in heart rate (from 66.5 [12.0] to 115.7 [22.2] beats min^−1^, *P*-value <0.0001) and a deferred decrease in stroke volume (from 61.3 [22.5] to 42.9 [14.8] ml, *P*-value <0.0001). For details, see Table 2 and Fig 1, Fig 2, Fig 3, Fig 4. Measurements after the Valsalva manoeuvre were too heterogeneous to be interpreted.

**Table 2:** Haemodynamic measurements before and after the manoeuvres. Mean and standard deviation (sd) of haemodynamic measurements before and after manoeuvres and respective *P*-values for changes between manoeuvres. T_0_=baseline before the start of the manoeuvre, T_1_=1 min after the completion of the manoeuvre (Valsalva: T_1_=30 s after start of manoeuvre), T_2_=5 min after the completion of the manoeuvre, T_end_=return to baseline. CO, cardiac output (L min^−1^), HR, heart rate (beats min^−1^); MAP, mean arterial pressure (mm Hg); PLR, passive leg raise; SV, stroke volume (ml).

Manoeuvre	Parameter	T_o_	T_1_	T_2_	T_end_
		Mean (sd)	Mean (sd)	Mean (sd)	Mean (sd)
**Stand- up**	SV (ml)	58.1 (19.2)	43.9 (17.1)	41.7 (13.7)	57.4 (23.6)
			*P*<0.0001 (T_1_*vs* T_0_)	*P*=0.3101 (T_2_*vs* T_1_)	*P*<0.0001 (T_end_*vs* T_2_) *P*=0.7605 (T_end_*vs* T_0_)
	CO (L min^−1^)	4.0 (1.4)	3.5 (1.5)	3.5 (1.3)	3.8 (1.6)
			*P*=0.0381 (T_1_*vs* T_0_)	*P*=0.8120 (T_2_*vs* T_1_)	*P*=0.1950 (T_end_*vs* T_2_) *P*=0.3073 (T_end_*vs* T_0_)
	MAP (mm Hg)	88.5 (9.0)	97.0 (9.6)	96.4 (8.8)	89.1 (8.0)
			*P*<0.0001 (T_1_*vs* T_0_)	*P*=0.6323 (T_2_*vs* T_1_)	*P*<0.0001 (T_end_*vs* T_2_) *P*=0.6463 (T_end_*vs* T_0_)
	HR (beats min^−1^)	69.0 (12.0)	81.3 (16.3)	84.1 (15.7)	66.3 (12.2)
			*P*<0.0001 (T_1_*vs* T_0_)	*P*=0.3050 (T_2_*vs* T_1_)	*P*<0.0001 (T_end_*vs* T_2_) *P*=0.3123 (T_end_*vs* T_0_)
**PLR**	SV (ml)	53.5 (19.5)	60.8 (23.2)	—	52.3 (20.4)
			*P*=0.0010 (T_1_*vs* T_0_)		*P*=0.5826 (T_end_*vs* T_0_)
	CO (L min^−1^)	3.6 (1.4)	4.1 (1.7)	—	3.4 (1.3)
			*P*=0.0130 (T_1_*vs* T_0_)		*P*=0.3777 (T_end_*vs* T_0_)
	MAP (mm Hg)	93.6 (9.0)	89.6 (9.9)	—	93.9 (8.4)
			*P*=0.0052 (T_1_*vs* T_0_)		*P*=0.8528 (T_end_*vs* T_0_)
	HR (beats min^−1^)	68.0 (11.9)	68.4 (12.2)	—	66.5 (12.3)
			*P*=0.8903 (T_1_*vs* T_0_)		*P*=0.5813 (T_end_*vs* T_0_)
**Step- test**	SV (ml)	57.5 (21.0)	61.3 (22.5)	42.9 (14.8)	60.1 (22.5)
			*P*=0.1770 (T_1_*vs* T_0_)	*P*<0.0001 (T_2_*vs* T_1_)	*P*<0.0001 (T_end_*vs* T_2_) *P*=0.2367 (T_end_*vs* T_0_)
	CO (L min^−1^)	3.8 (1.5)	7.0 (2.7)	4.0 (1.6)	4.0 (1.6)
			*P*<0.0001 (T_1_*vs* T_0_)	*P*<0.0001 (T_2_*vs* T_1_)	*P*=0.8774 (T_end_*vs* T_2_) *P*=0.1848 (T_end_*vs* T_0_)
	MAP (mm Hg)	88.9 (9.5)	110.5 (9.2)	97.0 (8.7)	89.8 (9.3)
			*P*<0.0001 (T_1_*vs* T_0_)	*P*<0.0001 (T_2_*vs* T_1_)	*P*<0.0001 (T_end_*vs* T_2_) *P*=0.5194 (T_end_*vs* T_0_)
	HR (min^−1^)	66.5 (12.0)	115.7 (22.2)	94.7 (24.4)	68.5 (13.0)
			*P*<0.0001 (T_1_*vs* T_0_)	*P*<0.0001 (T_2_*vs* T_1_)	*P*<0.0001 (T_end_*vs* T_2_) *P*=0.4438 (T_end_*vs* T_0_)
**Valsalva**	SV (ml)	58.3 (21.9)	50.7 (20.1)	—	56.8 (19.2)
			*P*=0,0006 (T_1_*vs* T_0_)		*P*=0.4940 (T_end_*vs* T_0_)
	CO (L min^−1^)	3.9 (1.6)	3.8 (1.6)	—	3.8 (1.5)
			*P*=0.6561 (T_1_*vs* T_0_)		*P*=0.5012 (T_end_*vs* T_0_)
	MAP (mm Hg)	89.8 (9.3)	100.7 (14.1)	—	89.0 (8.5)
			*P*<0.0001 (T_1_*vs* T_0_)		*P*=0.6185 (T_end_*vs* T_0_)
	HR (beats min^−1^)	67.9 (14.0)	76.3 (14.4)	—	66.9 (13.1)
			*P*=0.0022 (T_1_*vs* T_0_)		*P*=0.7131 (T_end_*vs* T_0_)

Perfusion index measurements were available for 18 subjects and are shown in Supplementary Table S1.

Associations between changes in PWTT (primary analyses) and changes in haemodynamic variables (secondary analyses) have been assessed using Pearson's correlation coefficient and are shown in Supplementary Table S2.

No significant correlation was found between the change in PWTT and the changes in cardiac output, stroke volume, mean arterial pressure, or heart rate.

## Discussion

In this prospective observational study, 24 healthy volunteers underwent various manoeuvres under noninvasive haemodynamic monitoring to study changes in the PWTT and to evaluate whether changes in PWTT reflected variations in intravascular fluid status. An increase in PWTT was observed during orthostasis induced by transitioning from a horizontal to an upright position. A decrease in PWTT was observed during the autotransfusion induced by the passive leg raise test. During an exercise stress test (step test), the PWTT initially remained unchanged with increasing cardiac output, indicating adrenergic activation without intravascular volume shift, but after 5 min, PWTT increased, most probably secondary to adrenergic activation with consequent vasoconstriction. Changes in the perfusion index were concordant with stroke volume responses, decreasing during orthostasis and the step test, while there was an increase during the passive leg raise, suggesting an influence of the manoeuvres performed on peripheral vascular tone. The results of the study support the hypothesis that PWTT shows promise to reflect immediate changes in intravascular volume. However, the clinical usefulness of PWTT to monitor intravascular fluid status deserves further studies, as PWTT seems to be influenced by more factors than preload changes alone.

Venous pooling causes the redistribution of 500–1000 ml of blood from the central compartment to the splanchnic capacitance vessels and lower extremities. Consequently, reduced venous return to the heart leads to a decrease in preload and induces a temporary relative hypovolaemia.^25^ During the ‘stand-up’ manoeuvre, 1 min after venous pooling, stroke volume and cardiac output significantly decreased, while PWTT increased. MAP and heart rate increased significantly, most likely due to reflective counterregulation. A similar PWTT response was observed in a study of 11 healthy individuals^8^ undergoing a tilt table test (head tilt 0–80°) to simulate central hypovolaemia. PWTT continuously increased with increasing tilt table angle. PWTT exhibited a stronger response to central volume loss than blood pressure at tilt table angles of 0–20°. This led to the conclusion that PWTT has the potential to be useful for the early detection of central hypovolaemia.

During the *passive leg raise test*, autotransfusion resulted in increased stroke volume and cardiac output, while PWTT decreased. Heart rate did not change significantly, and blood pressure decreased. No significant correlation was found between the change in PWTT and the changes in cardiac output, stroke volume, mean arterial pressure, or heart rate. The passive leg raise test simulates an increase in central blood volume of ∼300 ml from autotransfusion.^26^ Autotransfusion leads to an increase in ventricular filling and, consequently, to an increase in both stroke volume and cardiac output, as long as both ventricles operate on the steep part of the Frank–Starling curve. Concomitantly, PWTT decreases. In contrast to our study, a clinical study involving 30 patients after elective cardiac surgery (14 ventilated, six receiving dobutamine therapy, seven receiving norepinephrine therapy) did not observe any changes in PWTT after a modified passive leg raise test.^27^ Future studies are needed to explore the potential influence of ventilation or catecholamine/vasopressor therapy on the different PWTT responses observed after the passive leg raise test.

The *step test* had no significant impact on PWTT 1 min after the manoeuvre. Stroke volume, cardiac output, mean arterial blood pressure, and heart rate increased during exercise and returned to baseline levels after 5 min, with stroke volume falling below baseline after 5 min. Five minutes after completing the test, a significant increase in PWTT occurred. As the greatest change in PWTT was observed 5 min after recovery, rather than immediately after exercise, it can be concluded that PWTT is affected more strongly by vasoconstriction after an adrenergic stimulus (exercise) than by volume shifts. A retrospective study of 89 patients with cardiac disease who underwent cardiopulmonary exercise testing observed a decrease in PWTT during exercise,^28^ with a less pronounced decrease in heart failure patients, possibly because of their lower cardiac reserve.

Using the R-wave of the ECG and the earlobe for attaching the pulse oximeter was chosen for the following reasons: instead of using the Q-wave of the ECG for flow-based variables assessing fluid responsiveness in earlier studies,^6^^,^^7^ the use of the R-wave of the ECG has recently become standard.^8^^,^^9^^,^^27^, ^28^, ^29^, ^30^ No difference between Q-wave-based and R-wave-based PWTT measurements was shown in a clinical study with 38 patients undergoing elective intraabdominal surgery.^13^ As a more accurate reflection of central blood volume, the use of an ear pulse oximeter probe compared with a finger pulse oximeter probe might be more sensitive in detecting hypovolaemia.^13^^,^^31^ Thus, attaching the pulse oximeter at the earlobe for PWTT measurement seems to be less prone to errors induced by confounders such as hypothermia or the use of vasoactive medications.

Our study has several limitations. The evaluation of haemodynamic variables is lacking a true gold standard (i.e. electromagnetic flowmetry or cardiac magnetic resonance imaging). A ‘clinical gold standard’ such as transthoracic echocardiography cannot be considered a continuous monitoring technique. Additionally, transthoracic echocardiography is subject to intra-observer and inter-observer variability. To reduce these confounders, transthoracic echocardiography findings were assessed exclusively by two cardiac anaesthesiologists (EH and MD) with many years of experience. The intravascular volume status of study participants may have varied because participant fluid intake, caffeine/alcohol consumption, or exercising before the study was not standardised. Thus, volume responsiveness and the effects on PWTT and stroke volume in response to different manoeuvres might have been affected differently. Finally, it is currently unknown to what extent patient-specific factors such as age,^32^ size,^33^ the presence of hypertension,^34^ or peripheral arterial occlusive disease^35^ may influence PWTT itself. To minimise the impact of these factors, a rather homogeneous group of study participants (middle-aged adults without pre-existing disease) was recruited.

In summary, PWTT is capable of reflecting specific changes in haemodynamic status. A significant decrease in PWTT was observed during preload elevation (passive leg raise), while a significant increase in PWTT was observed during preload reduction (orthostasis). During exercise (step test), the PWTT initially remained unchanged, with an increase after 5 min. Stroke volume and cardiac output increased when PWTT decreased, and *vice versa*. The Valsalva manoeuvre is not a suitable manoeuvre for assessing haemodynamic changes in this study set-up.

For clinical practice, PWTT has potential as a noninvasive and cost-effective monitoring technique that could be particularly valuable as an early warning tool.

## Authors’ contributions

Contributed to the study conception and design: JMW, FG, KF, GP, IS

Acquisition and measurements: JMW, FG, KF, MD, EH, EW, EVG, GP

Statistical analyses: IS

Contributed to the interpretation of the data: all authors

Wrote the manuscript: JW, FG, KF, EVG, GP

Read, revised, and approved the final manuscript: all authors

## Declarations of interest

GP received an unrestricted educational grant from Nihon Kohden Europe GmbH, Rosbach, Germany. KF was involved in lectures on oesophageal Doppler monitoring organised by Deltex Medical Ltd., Chichester, UK. JMW received medical study equipment provided by Deltex Medical Ltd., Chichester, UK. EVG received payment for local lectures from Medtronic GmbH and Edwards Lifesciences Services GmbH (unrelated to the topic of this manuscript) and has no other financial or nonfinancial interests to disclose. FG, MD, EKH, IS, EW declare that they have no conflicts of interest.
