# Supplementary material for: Changes of pulse wave transit time after haemodynamic manoeuvres in healthy adults: a prospective randomised observational trial (PWTT volunteer study)
Source: BJA Open. 2024 Jun 24;11:100291. doi: 10.1016/j.bjao.2024.100291 (PMC11255098; doi:10.1016/j.bjao.2024.100291)
Supplement: Multimedia component 1 [file mmc1.docx]

Supplementary Table S1: Perfusion Index (PI) measurements before and after the manoeuvres

| **manoeuvre** | **T_o_** | **T_1_** | **T_2_** | **T_end_** |
| --- | --- | --- | --- | --- |
|  | **mean (SD)** | **mean (SD)** | **mean (SD)** | **mean (SD)** |
| **stand-up** | 6.28 (4.80) | 2.05 (2.13) | 1.85 (1.24) | 5.83 (4.37) |
|  |  | p<0.001 (T_1_ *vs.* T_0_) | p=0.7891(T_2_ *vs.* T_1_) | p<0.0001(T_end_ *vs.* T_2_)  p=0.5507(T_end_ *vs.*T_0_) |
| **PLR** | 3.46 (2.54) | 4.87 (4.37) | - | 3.91 (3.36) |
|  |  | p=0.0592(T_1_ *vs.* T_0_) | - | p=0.1967(T_end_ *vs.*T_1_)  p=0.5482(T_end_ *vs.*T_0_) |
| **step-test** | 4.81 (5.03) | 2.06 (1.46) | 2.55 (2.26) | 6.91 (4.96) |
|  |  | p=0.0003 (T_1_ *vs.* T_0_) | p=0.5105(T_2_ *vs.* T_1_) | p<0.0001(T_end_ *vs.* T_2_)  p=0.0051(T_end_ *vs.*T_0_) |
| **Valsalva** | 5.42 (5.38) | 3.16 (3.30) | - | 4.96 (4.71) |
|  |  | p=0.0025(T_1_ *vs.* T_0_) | - | p=0.0156(T_end_ *vs.* T_1_)  p=0.5373(T_end_ *vs.*T_0_) |

*mean and standard deviation (SD) of* Perfusion Index (PI) [%] measurements *before and after manoeuvres and respective p values for changes between manoeuvres. T_0_ = baseline before the start of the manoeuvre, T_1_ = 1 minute after the completion of the manoeuvre (Valsalva: T_1_ = 30 seconds after start of manoeuvre), T_2_ = 5 minutes after the completion of the manoeuvre, T_end_ = return to baseline.*

Supplementary Table S2: Correlations between haemodynamic changes and changes in PWTT-measurements

| **parameter** | **∆SV** | **∆CO** | **∆HF** | **∆MAP** |
| --- | --- | --- | --- | --- |
| stand-up | -0.0743 | -0.1143 | 0.0183 | -0.2377 |
|  | p=0.7300 | p=0.5950 | p=0.9325 | p=0.2634 |
| PLR | 0.1185 | 0.0983 | -0.1698 | -0.2702 |
|  | p=0.5813 | p=0.6476 | p=0.4277 | p=0.2016 |
| step-test | -0.2052 | -0.3527 | 0.1507 | 0.0270 |
|  | p=0.3597 | p=0.1074 | p=0.4822 | p=0.9002 |
| Valsalva | -0.0042 | -0.2421 | -0.4130 | 0.2534 |
|  | p=0.9845 | p=0.2543 | p=0.0449 | p=0.2322 |
| Partial correlation | -0.3967 | -0.3491 | -0.0060 | 0.0953 |
|  | p=0.0006 | p=0.0029 | p=0.9599 | p=0.4224 |

*Pearson correlation coefficient and respective p-values for correlations between changes in haemodynamic parameters and changes in PWTT-measurements. Changes between T0 = baseline before start of task and T1 = 1 minute after completion of task. ∆SV= change in stroke volume, ∆CO= change in cardiac output, ∆HF= change in heart frequency, ∆MAP= change in mean arterial pressure. Partial correlations were computed by partialling out participant.*
